# A snapshot of country-specific dietary habits and obesity in European children: the Feel4Diabetes study

**DOI:** 10.1007/s00431-025-06037-4

**Published:** 2025-02-27

**Authors:** Lubna Mahmood, Luis A. Moreno, Peter Schwarz, Ruben Willems, Greet Cardon, Soukaina Hilal, Imre Rurik, Violeta Iotova, Yuliya Bazdarska, Roumyana Dimova, Kostas Anastasiou, Yannis Manios, Esther M. Gonzalez-Gil, on behalf of the Feel4Diabetes-Study Group, Fernando Civeira, Gloria Bueno, Pilar De Miguel-Etayo, María L. Miguel-Berges, Natalia Giménez-Legarre, Paloma Flores-Barrantes, Aleli M. Ayala-Marín, Miguel Seral-Cortés, Lucia Baila-Rueda, Ana Cenarro, Estíbaliz Jarauta, Rocío Mateo-Gallego, Patrick Timpel, Timea Ungvari, Zoltán Jancsó, Anna Nánási, László Kolozsvári, Csilla Semánova, Éva Bíró, Emese Antal, Sándorné Radó, Remberto Martinez, Marcos Tong, Tsvetalina Tankova, Natalia Usheva, Kaloyan Tsochev, Nevena Chakarova, Sonya Galcheva, Yana Bachata, Zhaneta Radkova, Vanya Marinova, Tanya Stefanova, Kalliopi Karatzi, Odysseas Androutsos, George Moschonis, Spyridon Kanellakis, Christina Mavrogianni, Konstantina Tsoutsoulopoulou, Christina Katsarou, Eva Karaglani, Irini Qira, Efstathios Skoufas, Konstantina Maragkopoulou, Antigone Tsiafitsa, Irini Sotiropoulou, Michalis Tsolakos, Effie Argyri, Mary Nikolaou, Eleni-Anna Vampouli, Christina Filippou, Kyriaki Apergi, Amalia Filippou, Gatsiou Katerina, Efstratios Dimitriadis, Konstantinos Makrilakis, Stavros Liatis, George Dafoulas, Christina-Paulina Lambrinou, Angeliki Giannopoulou, Jaana Lindström, Tiina Laatikainen, Katja Wikström, Jemina Kivelä, Päivi Valve, Esko Levälahti, Eeva Virtanen, Tiina Pennanen, Seija lli, Karoliina Nelimarkka, Winne Ko, Ernest Karuranga

**Affiliations:** 1 Growth, Exercise, Nutrition and Development (GENUD) Research Group, University of Zaragoza, Zaragoza, Spain; 2 Centro de Investigación Biomédica en Red de Fisiopatología de La Obesidad y Nutrición (CIBERObn), Instituto de Salud Carlos III, Madrid, Spain; 3 Department for Prevention and Care of Diabetes, Medical Faculty Carl Gustav Carusat Theaq, Technical University of Dresden, Dresden, Germany; 4 Paul Langerhans Institute Dresden of the Helmholtz Center Munich at University Hospital and Faculty of Medicine, TU Dresden, 01307 Dresden, Germany; 5 German Center for Diabetes Research (DZD E.V.), 85764 Neuherberg, Germany; 6 Department of Public Health and Primary Care, Ghent University, Ghent, Belgium; 7 Department of Movement and Sports Sciences, Ghent University, Ghent, Belgium; 8 Doctoral School of Health Sciences, University of Debrecen, Debrecen, Hungary; 9 Department of Family Medicine, Semmelweis University, Budapest, Hungary; 10 Department of Pediatrics, Medical University of Varna, Varna, Bulgaria; 11 Division of Diabetology, Department of Endocrinology, Medical University of Sofia, Sofia, Bulgaria; 12 Department of Nutrition and Dietetics, School of Health Science & Education, Harokopio University, Athens, Greece; 13 Institute of Agri-Food and Life Sciences, Hellenic Mediterranean University Research Centre, Heraklion, Greece; 14 Instituto Agroalimentario de Aragón (IA2), Zaragoza, Spain; 15 Instituto de Investigación Sanitaria de Aragón (IIS Aragón), Zaragoza, Spain

**Keywords:** Children, Dietary intake, Food frequency, Obesity, Europe

## Abstract

This study assessed the dietary intake of European children by country, gender, and obesity degree across six European countries participating in the Feel4Diabetes study. It also compared food group intakes with European food-based dietary guidelines. The Feel4Diabetes study included 9,847 children (50.4% girls) from six European countries. Families provided baseline data on energy balance-related behaviors and socioeconomic factors via questionnaires. Children’s dietary intake and breakfast consumption were assessed using semi-quantitative food frequency questionnaires, and anthropometric measurements were taken. Intake from 11 food groups was analyzed by country, gender, and obesity degree. Chi-square analyses compared food intake with dietary guidelines. “Nutrient-dense " foods (e.g., fruits, vegetables, whole grains) and "energy-dense, low-nutrient" foods (e.g., high in added sugars, saturated fats) were categorized based on the European food-based guideline. Over 85% of children consumed breakfast 5–7 days a week, with no significant differences by Body Mass Index (BMI) or country. Mean intakes of nutrient-dense food groups fell below recommendations, while the intake of energy-dense, low-nutrient foods exceeded limits, particularly in children with overweight/obesity (*p* = 0.048). No significant gender differences were observed in most food groups.

*Conclusions*: Dietary intake varied by country and obesity degree among European children. Across the six countries, children’s diets did not align with food-based dietary guidelines. These findings underscore the need for interventions promoting healthier eating habits among European children.

*Trial registration*: The Feel4Diabetes-study is registered with the clinical trials registry (NCT02393872), http://clinicaltrials.gov

**Table:** 

**What is Known:**	
• *Childhood obesity is a growing concern across Europe, with some variations in food consumption observed between European countries*	
• *Previous studies have highlighted the role of diet in childhood obesity, but few have examined cross-country comparisons while accounting for gender and BMI status.*	
**What is New:**	
• *This study provides a comprehensive cross-country analysis of children's dietary intake, considering gender, BMI status, and adherence to European dietary guidelines.*	
• *New insights into gender differences in sweets consumption and the positive association between BMI and food intake (such as soft drinks and sweets) are presented, which can inform targeted dietary interventions.*

**Supplementary Information:**

The online version contains supplementary material available at 10.1007/s00431-025-06037-4.

## Introduction

Healthy eating is critical for preventing non-communicable diseases and malnutrition [1]. Modern diets high in refined carbohydrates, sugar, and trans fats have contributed to rising childhood obesity rates [1]. The WHO European Childhood Obesity Surveillance Initiative (2015–2017) found that 29% of children aged 7–9 were overweight, with boys (31%) more affected than girls (28%) [2]. Childhood obesity increases the risk of non-communicable diseases and often persists into adulthood [3]. Regional variations exist, with Cyprus, Spain, Italy, and Greece having the highest rates, while Denmark reported the lowest [2]. Obesity results from genetic, behavioral, and environmental factors, with an imbalance between energy intake and expenditure central to weight gain [3]. Excess consumption of energy-dense, low-nutrient foods exacerbates this issue [4].

The concept of a healthy diet has evolved, emphasizing balanced macronutrients, fruits, vegetables, whole grains, nuts, and legumes, while limiting salt, sugars, saturated fats, and processed foods [5]. Traditional diets like the Mediterranean diet promote health with more healthful foods [5]. Breakfast skipping has been linked to poor dietary quality and increased obesity risk, while regular breakfast consumption supports weight management [6].

Dietary habits vary across Europe [7–9]. The IDEFICS study (16,228 children, 2–9 years) found Italy had the highest Mediterranean-like diet adherence, while Spain and Cyprus had the lowest [7]. The WHO’s fourth childhood obesity surveillance reported low savory snack consumption in Northern Europe and lower vegetable intake in Western Asia, particularly among boys [8]. Dietary patterns are influenced by socioeconomic, cultural, and environmental factors, including food availability, traditions, and national guidelines [10].

Understanding food composition is essential for informed dietary choices [11]. The European Food-Based Dietary Guidelines (FBDG) offer evidence-based recommendations to prevent nutrient deficiencies and obesity [12]. This study aims to assess children's dietary intake across six European countries, examining variations by country, gender, and obesity status while evaluating adherence to the FBDG to inform public health strategies.

## Methods

### Study design

The Feel4Diabetes study aimed to combat obesity and related comorbidities by promoting a healthy lifestyle in 11,396 families across six European countries. Based on the world bank country classification derived from the 2013 Gross National Income per capita [13], the countries in the study were grouped in regions as: high-income countries (Finland and Belgium), countries under austerity measures following the economic crisis (Spain and Greece), and low-income countries (Hungary and Bulgaria). In Feel4diabetes study, the “vulnerable areas” were defined as every area in low-income countries and countries under austerity measures, and only low socioeconomic status areas in high-income countries. Children in the first three primary school grades and their parents were invited to participate. Data were collected at baseline (2016) and during follow-ups in 2017 and 2018. This analysis uses the baseline cross-sectional data only. The study is registered at http://clinicaltrials.gov (NCT02393872), and the full protocol has been published [14]. Ethical approval was obtained in each country, and all participants provided written informed consent before enrolment.

### Study sample

At baseline in the Feel4Diabetes-study, 11,396 families were included. Children’s information was reported by the parent with full-filled questionnaire along with completed anthropometric measures. Since some families included more than one child, we randomly selected one child per family in order not to duplicate parental information, one child from each family was included and linked to the reported parental information. Among the 11,396 families meeting the inclusion criteria, 1,549 children were excluded because of incomplete data and missing anthropometric measurements, resulting in 9,847 children being included in the study.

### Dietary assessment

A semi-quantitative food frequency and eating behavior questionnaire was provided to families, completed by one parent at home. The questionnaire was adapted from the Finnish diabetes prevention program (FIN-D2D) [13] and modified culturally for the six European countries in the study. Initially developed in English, the questionnaire was translated into each participating country’s language, followed by back-translation into English and fine-tuning for local nuances. The child-specific version of the questionnaire mirrored the parents’ version but excluded alcohol and coffee-related questions.

To ensure reliability, the test–retest reliability was assessed using intra-class correlation coefficients (ICC) in 191 parent–child pairs, who completed the questionnaires on two occasions with a 1–2 week interval [13].

The study assessed children’s consumption of various food items, including breakfast, grains, fruits, vegetables, legumes, red meat, poultry, fish, dairy products, savory snacks (e.g., croissants, cheese pies), sweets (e.g., pancakes, cookies), and soft drinks. Breakfast consumption was measured by asking, "On how many days does your child usually eat breakfast?" with options ranging from "never" to "daily." For dietary intake, parents reported the frequency of their child’s consumption of each food item, using a scale from “less than one serving per week” to “5 or more servings per day.” Portion sizes were provided in household units, such as one medium apple (90 g) or half a cup of chopped fruit.

The terms 'nutrient-dense’ foods and 'energy-dense, low-nutrient' foods were defined by comparing participants' dietary intake to the FBDG [12]. 'Nutrient-dense' foods align with FBDG recommendations, including fruits, vegetables, whole grains, and lean proteins, while ' energy-dense, low-nutrient' foods deviate from these recommendations, such as those high in added sugars, saturated fats, and low in essential nutrients. Although the FBDGs are largely consistent across countries, minor discrepancies may exist. A detailed list of foods categorized as 'nutrient-dense’ and 'energy-dense, low-nutrient' according to these guidelines is provided in the supplementary material (Supplementary Table 1).

### Anthropometric measurements

Children’s height and weight were measured at schools by trained researchers following standardized procedures [14]. Body weight, measured in light clothing and barefoot using a Seca 813 digital scale, was recorded to the nearest 0.1 kg. Height was measured with a Seca 217 stadiometer, with the head in the Frankfurt plane, and recorded to the nearest 0.1 cm. Two readings were taken for each measurement, with a third taken if differences exceeded 100 g for weight or 1 cm for height. The average of the two closest readings was used for analysis. BMI was calculated as weight (kg) divided by height (m^2^), and children were categorized into underweight, normal weight, and overweight/obesity. Age- and gender-specific BMI z-scores (zBMI) were calculated following Cole cut-offs [15].

### Statistical analysis

Descriptive data are presented as percentages for categorical variables and means for continuous variables. The Kolmogorov–Smirnov test assessed variable distributions. Gender interactions in dietary habits and weight status were observed, so analyses were stratified by gender. Student’s t-tests compared continuous variables, and Pearson’s chi-square test was used for categorical variables. Results were stratified by country, gender, and BMI categories. Food consumption frequencies from 11 food groups were compared to the FBDGs [12] (see Supplementary File 1). To handle the Missing at Random (MAR) data, multiple imputation (MI) was performed using a fully conditional specification method. A total of 150 cases (1.5%) were imputed across several variables. The imputation model included all relevant covariates, and the resulting 20 imputed datasets were analyzed. Sensitivity analyses were then conducted to compare results from the imputed and complete case analyses to assess the robustness of the findings. Bonferroni test was used when making multiple comparisons. A complete-case analysis was used for transparency. Figures were created using Microsoft Excel (version 16.79.2), and statistical analyses were performed using IBM-SPSS (Version 26.0), with *p* < 0.05 considered statistically significant.

## Results

Out of 11,396 families that met the inclusion criteria, 1,549 children were excluded for incomplete information and lack of anthropometric measurements, and 9,847 were included in this study, data were missing for approximately 150 (1.5%) cases due to random missingness across various variables. A total of 9,847 children were analyzed, with participants ranging from 2,609 in Bulgaria to 1,211 in Finland. The majority of participants were 7 years old (67%), with 50.4% being girls. Additionally, 8% were 6 years old, 13% were 8 years old, and 12% were 9 years old.

Overweight/obesity rates were highest in Greece (36.5%) and Hungary (35.5%), with Belgium having the lowest (13.2%) (Table 1). Most children (92.2%) ate breakfast 5–7 days per week, while 2.6% rarely did. Daily fruit consumption was reported by 38%, while 11.8% rarely ate it. Daily vegetable intake was 26.5%, with 19.2% consuming them infrequently. Sweets were consumed daily by 16.1%, savory snacks by 4.9%, and soft drinks by 8.7%.

**Table 1:** The percentage (%) of children (6–9 years) participating by gender, and country (*n* = 9,847)

Country	Children total (%)	Percentage of children participating by gender (%)		BMI categories (%)		
		Boys (%)	Girls (%)	Underweight	Normal	Overweight/obesity
Belgium	14.7	49.7	50.3	2.6	84.2	13.2
Finland	12.3	50.2	49.8	3.2	64.1	32.7
Greece	18.7	47.8	52.2	2.4	61.1	36.5
Spain	13.9	52.8	47.2	5.2	72.4	22.4
Hungary	13.9	47.8	52.2	5.1	59.3	35.5
Bulgaria	26.5	48.8	51.2	6.8	65.3	27.9

### Differences by country and BMI

#### Meal occasion—breakfast

**Fig. 1:**
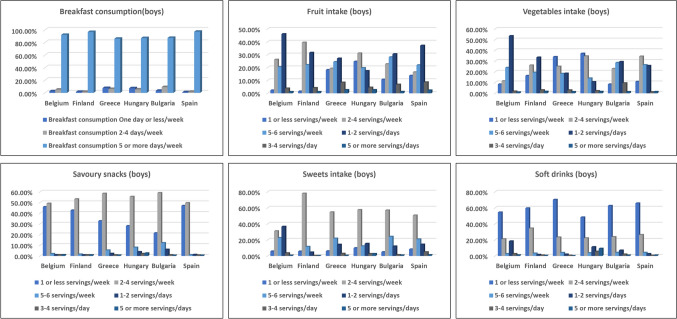
Frequency of consuming breakfast, fruit, and vegetables, savoury snacks, sweets, and soft drinks among boys by country

The frequency of breakfast consumption among European children (boys/girls) is shown in Figs. 1 and 3. Breakfast consumption (5–7 days per week) ranged from 86.1% in Greece to 97.2% in Spain, with no significant differences between countries (*p* = 0.23) or genders. Breakfast intake across BMI categories was consistent. Among boys, 53.2% in the overweight/obesity group and 60.0% in the underweight group consumed breakfast regularly (*p* = 0.824). Similarly, for girls, 52.3% in the overweight/obesity group and 62.7% in the normal weight group ate breakfast regularly, with no significant differences (*p* = 0.335).

#### Consumption food groups: fruits, vegetables, legumes and grains

Daily fresh fruit consumption varied significantly between regions (*p* < 0.05), with Hungary at 17.4%, Bulgaria at 30.5%, and Belgium at 46.1% (Figs. 1 and 3). Children in Finland had the lowest percentage consuming fruit "never or once a week" (1.5%), while Hungary had the highest (24.7%). No significant gender differences were observed. Among boys with overweight/obesity, 36.3% consumed 5–6 servings per week, compared to 27.7% of normal-weight boys (*p* = 0.330). Girls with overweight/obesity consumed 5–6 servings per week at 35.2%, with no significant differences (*p* = 0.581).

**Fig. 2:**
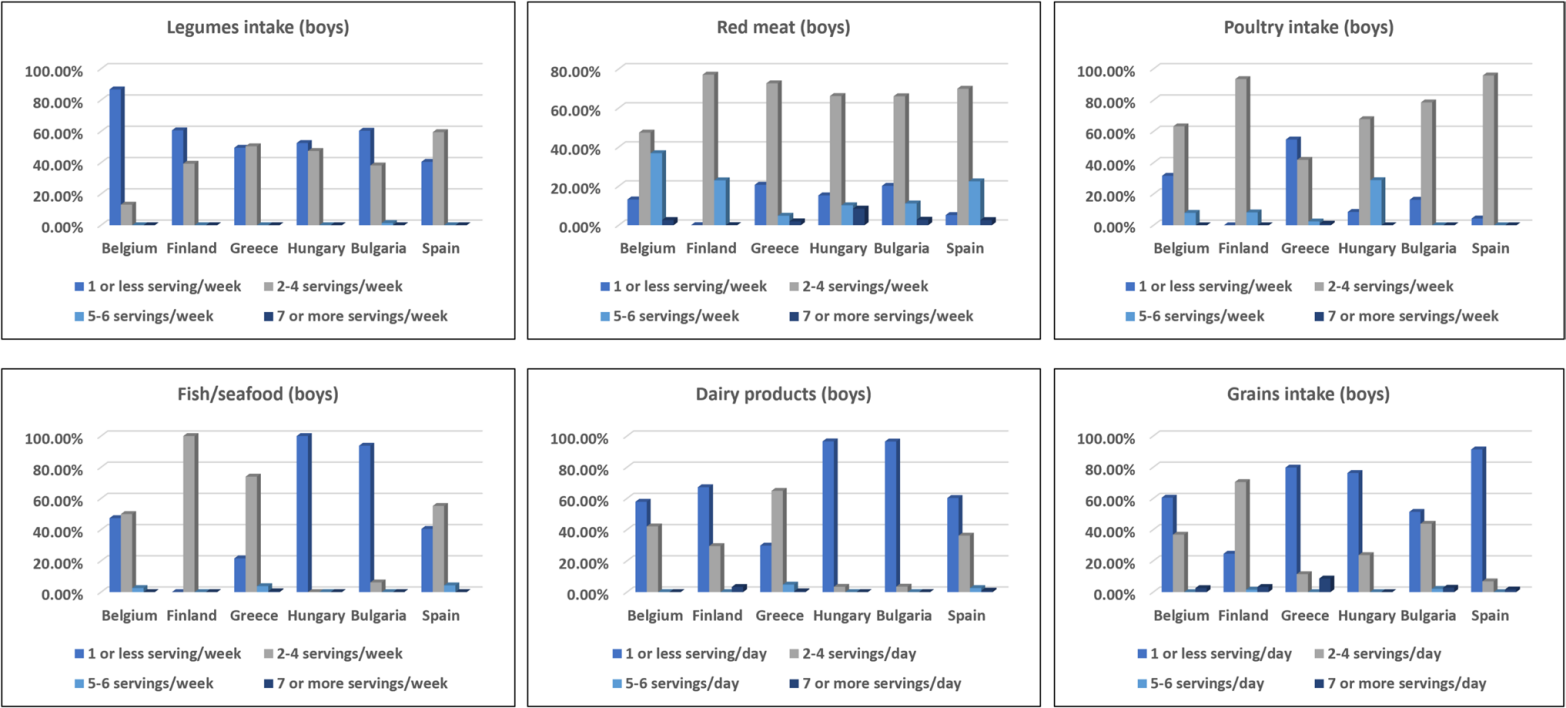
Frequency of consuming legumes, red meat, poultry, fish/seafood, dairy products, and grains among boys by country

Daily vegetable consumption ranged from 10.6% in Hungary to 53.5% in Belgium. Higher intake "never or once a week" was observed in Hungary (37.1%) and Greece (34.0%) compared to Belgium (8.4%) and Bulgaria (8.2%) (Figs. 2 and 4). Girls tended to consume vegetables more frequently than boys. Boys with overweight/obesity showed a trend toward higher intake, with 34.7% consuming 5–6 servings per week (*p* = 0.095), while girls had the highest intake (35.5%), with no significant differences (*p* = 0.233). Legume intake was lowest in Belgium and Finland.

#### Consumption of food groups: red meat, poultry, fish/seafood, and dairy products

Boys in Finland and girls in Greece had the highest moderate red meat intake (77.05% and 75%), while boys in Belgium and Spain consumed 720–840 g/week. Girls in Hungary and Bulgaria showed more varied intake, with significant percentages consuming 5–6 servings weekly. Red meat consumption did not significantly vary by BMI (*p* > 0.05). Poultry intake was higher among boys in Hungary and Bulgaria (2–4 servings/week), with Finland and Greece leading overall intake. Overweight boys (28.8%) and girls consumed 7 + servings/week, with significant BMI-related differences (boys: *p* = 0.021, girls: *p* = 0.039). Fish/seafood intake was highest in Finland and Greece (74%) and lowest in Hungary and Bulgaria (1% and 6.4%), with no significant BMI differences (*p* > 0.05). Dairy intake was low across countries, particularly among boys in Hungary and Bulgaria (96.6% and 96.5%) (Figs. 2 and 4).

#### Consumption of sweets, soft drinks and savoury snacks

**Fig. 3:**
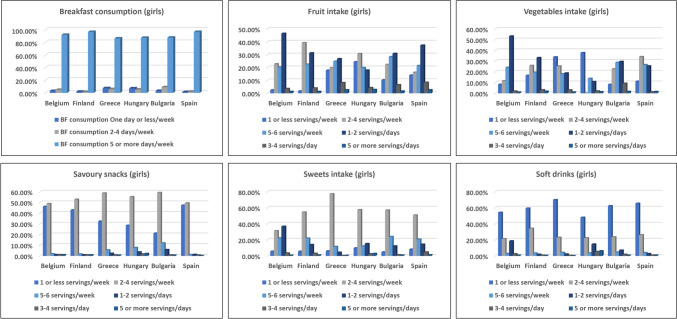
Frequency of consuming breakfast, fruit, and vegetables, savoury snacks, sweets, and soft drinks among girls by country

Figures 1 and 3 show significant variation in sweet snack consumption across countries, with daily intake ranging from 4.5% in Finland to 36.6% in Belgium. The percentage of children who consumed sweets rarely or never varied from 4.9% in Bulgaria to 9.9% in Hungary.

Boys and girls of normal weight reported similar frequencies of sweet intake, but no significant differences were observed. Soft drink consumption was lowest in Finland (1.6%) and Greece (2.0%) and highest in Belgium (18.4%) and Hungary (11.2%). Fewer children in Hungary consumed soft drinks weekly or less compared to Greece. Daily soft drink intake was higher among boys and girls with overweight/obesity, with significant differences by BMI (Table 2).

**Table 2:** Dietary intake of European children (*n* = 9,847), by gender and obesity degree

Dietary intake		Boys (%)			*p*-value	Girls (%)			*p*-value
		BMI category				BMI category			
		Underweight	Normal weight	Overweight/obesity		Underweight	Normal weight	Overweight/obesity	
Breakfast consumption	One day or less/week	20.0	13.6	15.7	0.824	25	10.8	17.2	0.335
	2–4 days/week	20.0	27.1	31.1		18.0	26.5	30.5	
	5 or more days/week	60.0	59.3	53.2		57.0	62.7	52.3	
Fruit intake	1 or less serving/week	3.0	8.5	9.5	0.330	2.5	9.3	8.1	0.581
	2–4 servings/week	56.0	35.7	22.1		55.0	34.2	23.8	
	5–6 servings/week	16.0	20.1	36.3		15.0	21.4	35.2	
	1–2 servings/day	11.0	27.7	24.9		12.0	27.8	25.4	
	3–4 servings/day	12.0	6.5	4.6		10.5	6.7	4.9	
	5 or more servings/day	2.0	1.5	2.6		5.0	0.6	3.6	
Vegetables	1 or less serving/week	4.0	2.0	1.5	0.095	3.5	1.5	1.0	0.233
	2–4 servings/week	28.0	30.6	36.8		27.0	31.0	37.2	
	5–6 servings/week	24.0	24.3	34.7		26.0	24.5	35.5	
	1–2 servings/day	32.0	28.7	17.7		30.0	28.5	18.0	
	3–4 servings/day	8.0	6.8	5.7		9.0	7.5	5.0	
	5 or more servings/day	4.0	7.6	3.6		4.5	7.0	2.3	
Savory snacks	1 or less serving/week	52.0	57.0	55.2	0.148	50.0	55.0	57.5	0.080
	2–4 servings/week	41.0	39.1	37.3		42.0	38.0	35.0	
	5–6 servings/week	1.0	2.6	4.7		2.0	2.0	5.0	
	1–2 servings/day	4.0	0.9	1.5		4.0	1.0	1.5	
	3–4 servings/day	1.0	0.2	0.5		1.0	0.5	0.5	
	5 or more servings/day	1.0	0.2	0.8		1.0	0.5	0.5	
Sweets	1 or less serving/week	8.0	4.8	4.7	0.052	9.0	4.5	4.0	0.074
	2–4 servings/week	48.0	58.8	54.2		47.0	57.5	53.0	
	5–6 servings/week	24.0	19.2	23.6		25.0	18.5	24.5	
	1–2 servings/day	12.0	14.5	14.7		12.0	15.0	15.5	
	3–4 servings/day	4.0	1.4	1.5		4.0	2.0	2.5	
	5 or more servings/day	4.0	2.2	1.3		3.0	2.5	0.5	
Soft drinks	1 or less serving/week	4.0	8.8	4.8	**0.048**	3.0	9.0	5.0	**0.021**
	2–4 servings/week	18.5	40.9	11.2		17.0	41.0	11.5	
	5–6 servings/week	12.0	11.8	28.1		11.0	12.0	27.0	
	1–2 servings/day	42.1	18.7	17.8		43.0	18.0	18.0	
	3–4 servings/day	20.0	15.0	32.0		22.0	14.0	31.5	
	5 or more servings/day	3.4	4.8	6.1		4.0	2.0	7.0	
Legumes	1 or less serving/week	5.5	10.3	15.7	0.242	6.5	11.7	17.9	0.178
	2–4 servings/week	55.2	40.6	34.8		54.5	40.1	34.5	
	5–6 servings/week	24.7	30.2	25.5		25.1	30.7	26.0	
	7 or more servings/week	14.6	18.9	24.0		13.9	17.5	21.6	
Red meat	1 or less serving/week	6.2	10.8	14.3	0.086	7.0	12.1	16.0	0.073
	2–4 servings/week	49.8	38.3	26.1		49.2	37.9	25.9	
	5–6 servings/week	21.8	30.4	32.0		22.1	30.9	32.6	
	7 or more servings/week	22.2	20.5	27.6		21.7	19.1	25.5	
Poultry	1 or less serving/week	4.3	11.9	15.1	**0.021**	5.5	12.1	15.4	**0.039**
	2–4 servings/week	48.5	37.3	26.2		49.0	37.8	26.5	
	5–6 servings/week	26.1	28.4	29.9		26.4	28.7	30.3	
	7 or more servings/week	21.1	22.4	28.8		19.1	21.4	27.8	
Fish/seafood	1 or less serving/week	7.3	13.7	16.4	0.058	7.9	14.5	17.4	0.067
	2–4 servings/week	51.8	41.7	28.9		52.0	41.9	29.0	
	5–6 servings/week	19.8	24.3	30.1		20.3	25.1	31.1	
	7 or more servings/week	21.1	20.3	24.6		19.8	18.5	22.5	
^1^ Dairy products	1 or less serving/day	10.5	15.2	19.8	0.091	11.4	16.4	21.4	0.096
	2–4 servings/day	49.7	45.3	35.1		50.6	46.2	35.7	
	5–6 servings/day	24.8	25.1	30.1		24.9	25.3	30.5	
	7 or more servings/day	15.0	14.4	15.0		13.1	12.1	12.4	
^2^ Grains	1 or less serving/day	8.5	12.3	18.2	0.141	9.4	13.6	20.1	0.098
	2–4 servings/day	41.9	38.6	30.3		42.7	39.4	31.0	
	5–6 servings/day	30.2	35.4	31.9		31.0	36.3	32.6	
	7 or more servings/day	19.4	13.7	19.6		17.0	10.7	15.8	

*N* = 9,847 children. *P* < 0·05 (Bold indicate significance). ^1^ cheese was not counted. ^2^ (rice and pasta were not mentioned under Grains group in the questionnaire). 1 serving of dairy = ½ cup (120 mL), 1 serving of grains = a roll (60 g) equals 2 slices of bread, 1 serving of fruit = 90 gm (1 medium sized apple/2 small apricots), 1 serving of vegetables = ½ cup of vegetables or at the size of a tennis ball tomato, broccoli or leafy, 1 serving of legumes = ½ cup (100 g), 1 servings of red meat/poultry/fish = 1 pack of cards (100–120 g), 1 serving of soft drinks = 1 glass (250 mL), 1 servings of salty snacks = 1 small bag of chips (100 g), 1 slice of pizza (160 g), 1 serving of sweets = 1 small chocolate bar (40 g), 1 cookie, 4 pieces of chocolates, or 1 scoop of ice cream. The numbers in the table are presented as percentages

Savory snack consumption was lowest in Finland (0.9%), Belgium (1.0%), and Spain (1.2%), and was high across all BMI categories with no significant gender differences.

### Food recommendation results

**Fig. 4:**
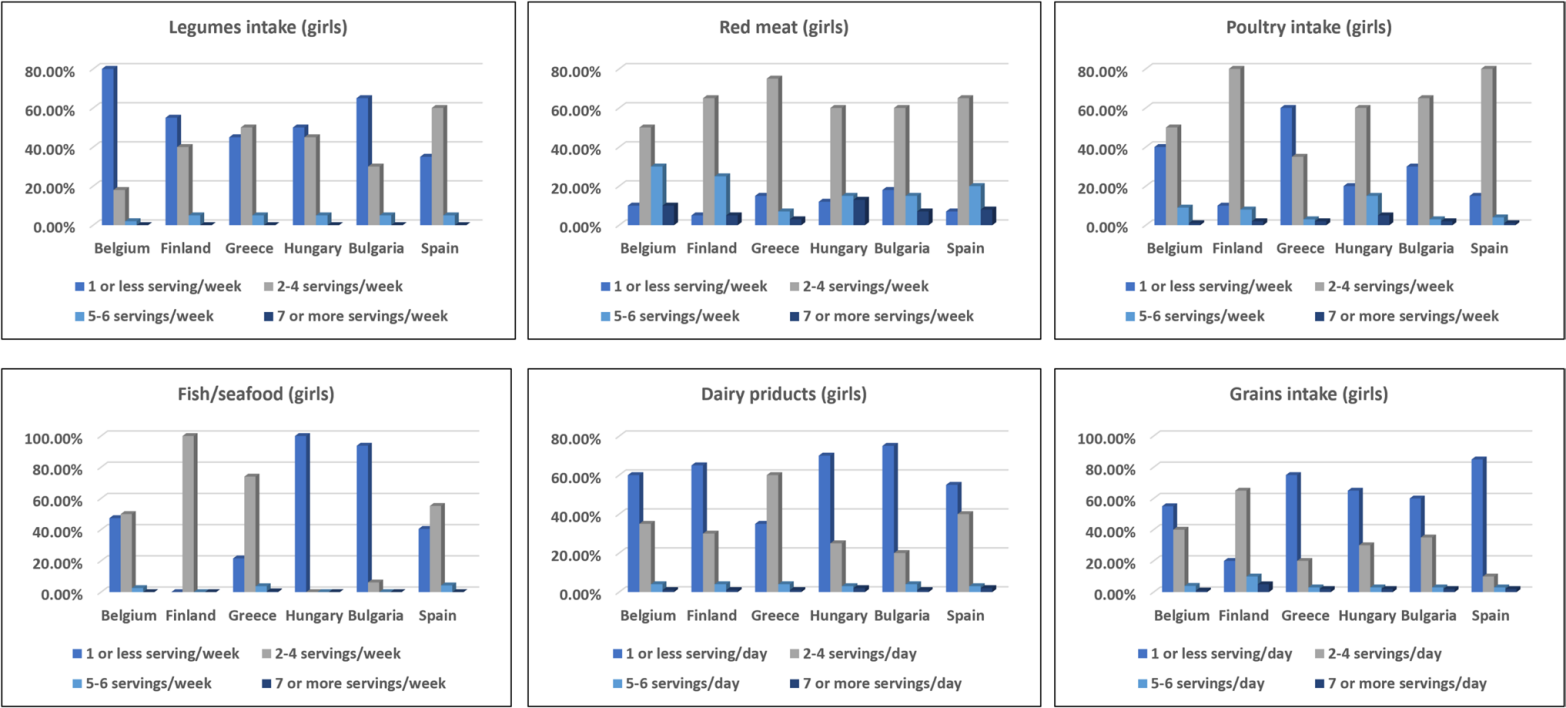
Frequency of consuming legumes, red meat, poultry, fish/seafood, dairy products, and grains among girls by country

A majority of children did not meet the recommended servings of fruits, vegetables, fish, or grains. Most consumed 1–4 servings of FV per week, far below the 2–3 servings/day guideline. In Finland, 80% met fish/seafood recommendations, but most children fell short in other countries. Grain and dairy intake also lagged behind recommendations, while red meat, poultry, sweets, soft drinks, and savory snacks exceeded recommended limits, reflecting suboptimal dietary habits across food groups (Figs. 1, 2, 3, and 4).

## Discussions

This study offers novel insights into the dietary habits of primary-school-aged children across six European countries, providing a comprehensive analysis of dietary intake by country, gender, and obesity status. Unlike previous studies, which often focused on individual countries or specific food items, our research examines food group consumption and adherence to European dietary recommendations in a cross-country context. Key findings include significant gender differences in sweets consumption and positive BMI-related associations in soft drink intake, particularly among boys with overweight/obesity. These results highlight the need for targeted interventions to address these dietary disparities. This study aligns with previous research, like the IDEFICS study, highlighting varied dietary intake influenced by social, cultural, and economic factors [7, 10, 16].

### Differences by country and BMI

#### Meal occasion – breakfast

Our study found that over 97% of Spanish children have breakfast five to seven days a week, with no significant gender difference, consistent with previous European research [17, 18]. A cross-sectional study across 33 countries reported higher breakfast skipping rates (10%−30%), particularly among teenagers, especially females [18]. Family structure and parental modeling significantly influence breakfast consumption, with children in single-parent families more likely to skip meals [19, 20]. Unlike some studies [21], we found no gender differences, contrasting with Italian data showing girls and children with overweight or obesity were more likely to skip breakfast [22]. Skipping meals is linked to unhealthy eating habits and higher obesity rates [23].

#### Consumption of food groups: fruits, vegetables, legumes and grains

Improving children’s diets relies on increasing fruit and vegetable (FV) consumption. Our study found low FV intake among Bulgarian children, with significant variations across European countries. Seasonal availability may influence these differences, as data collection occurred during potentially lower FV accessibility periods [24]. Socioeconomic factors, including education and income, also play a critical role in shaping dietary habits [24].

Legume consumption was low among children in Belgium and Finland, with many eating one or fewer servings per week. Girls in Spain, Greece, and Hungary showed a higher tendency for moderate legume intake compared to boys. Interventions like Beans4Life [25] in Portugal and SmartFeeding4Kids [26] across Europe have highlighted inadequate knowledge and infrequent consumption of legumes, reflecting low health literacy. Despite their nutritional benefits, legumes are often replaced by increased meat consumption [25, 26].

Our study found that girls generally consumed more grains than boys, consistent across countries, with no significant differences in FV, legume, and grain consumption by BMI categories [27–29].

#### Consumption of food groups: red meat, poultry, fish/seafood, and dairy products

This research shows that boys and girls in Finland and Greece have the highest moderate consumption of red meat, poultry, and fish/seafood, while Spain has notably higher red meat intake. Our findings are similar to those reported in the literature highlighting Spain’s high animal protein density, particularly from red meat and dairy [16, 30]. Cultural and geographical factors influence consumption patterns, with Finland’s abundant freshwater and coastline contributing to high fish consumption [10, 31].

Most children consumed one or fewer servings of dairy daily, with consistent gender patterns; however, Spain showed more balanced consumption. Spanish preschoolers consume more dairy than peers in Germany and Hungary [16], reflecting Mediterranean dietary intake [32]. No notable differences were found in intake across BMI categories.

#### Consumption of sweets, soft drinks and savoury snacks

Our data revealed daily sweet consumption ranged from 4.5% in Finland to 36.6% in Belgium, with Belgium leading the EU in sweet and chocolate intake. High sweet consumption is linked to obesity risk, as noted in studies from Greece and Ontario [33, 34]. Children's early preferences for sugary foods significantly influence long-term dietary habits, emphasizing the need for early intervention to promote healthier eating patterns [35].

Daily soft drink consumption varied from 1.6% in Finland to 18.4% in Belgium, consistent with a WHO study showing significant between-country differences [36]. Socio-economic factors affect these habits, with higher parental occupational status associated with lower soft drink consumption in Northern, Southern, and Western Europe.

Our results indicated that 6.0% of Bulgarian, 3.8% of Hungarian, and 2.2% of Greek children consumed 1–2 servings of savory snacks daily, with no significant variations by country or gender. Notably, children with overweight/obesity showed increased soft drink consumption compared to those with normal weight, aligning with findings from the INMA Project [37] and other studies [38, 39].

#### Children’s actual intake Vs European Food-based Dietary Guidelines

Our results showed that most children consume only 1 to 4 servings of fruits and vegetables (FV) per week, well below the recommended 2 to 3 servings daily [40]. Factors such as parents' eating habits, FV availability, cost, and lack of nutritional knowledge contributed to this low intake. Grain consumption was also below the recommended 3 daily servings, partly due to study methodology and limited food categories in the FFQ. The Dutch study found most children met or exceeded grain recommendations, primarily from refined grains [41].

Our FFQ excluded items like "rice" and "pasta," likely underestimating grain intake. Insufficient legume intake was observed, consistent with findings from North America, Europe, and Oceania [16]. Fish and seafood intake was generally low, except in Finland, where over 80% met recommendations [10, 31]. Dairy consumption averaged 2–4 servings per week, instead of the recommended 2–3 per day, consistent with declining milk intake in Germany [42]. Excessive sweets and soft drinks were common [33, 43].

#### Limitations and strengths

The study’s cross-sectional design and reliance on parental self-reported food data may limit accuracy, as self-reported questionnaires are less precise than methods like 24-h dietary recalls [44, 45]. Social desirability bias may also affect results, as participants might report dietary intake aligning with perceived healthy eating norms. The uneven distribution of participants across countries limits the generalizability of findings, introducing potential bias. Despite these limitations, the study’s strength lies in its large, diverse cohort from six European countries, with standardized procedures and trained researchers ensuring the accuracy of anthropometric measurements.

## Conclusion

Our study highlights diverse dietary habits among primary-school-aged children in Europe, with significant variations in food intake by country, gender, and obesity status. 'Nutrient-dense’ foods consumption generally fell below recommendations, while 'energy-dense, low-nutrient' food, particularly in overweight/obese children, exceeded limits. These patterns are influenced by environmental, parental, cultural, and socioeconomic factors. Future research should explore these relationships further to develop targeted, context-specific interventions. Collaborative efforts among healthcare professionals, educators, and policymakers are essential to improve children's nutrition and reduce the prevalence of childhood obesity.

## Supplementary Information

Below is the link to the electronic supplementary material.

ESM 1 (DOCX 19.7 KB)

## Data Availability

Data is provided within the manuscript.

## Abbreviations

BMI: Body mass index
FBDG: Food-based dietary guidelines
FFQ: Food frequency questionnaire
FV: Fruit and vegetables
FIN-D2D: Finnish diabetes prevention program
ICC: Intra-Class Correlation coefficients
zBMI: BMI z-scores

## References

[1] Allman-Farinelli M (2023) Nutritional strategies to prevent weight gain and obesity. Nutrients 15(19):4180 37836463 10.3390/nu15194180 PMC10574331

[2] WHO European Childhood Obesity Surveillance Initiative (COSI) (2018–2020) Report on the fifth round of data collection. Available from https://www.who.int/europe/publications/i/item/WHO-EURO-2022-6594-46360-67071. Accessed 23 June 2024

[3] Lister NB, Baur LA, Felix JF, Hill AJ, Marcus C, Reinehr T, Summerbell C, Wabitsch M (2023) Child and adolescent obesity. Nat Rev Dis Primers 9(1):24 10.1038/s41572-023-00435-4 37202378

[4] Fruh SM (2017) Obesity: risk factors, complications, and strategies for sustainable long-term weight management. J Am Assoc Nurse Pract 29:S3–S14 29024553 10.1002/2327-6924.12510 PMC6088226

[5] Cena H, Calder PC (2020) Defining a healthy diet: evidence for the role of contemporary dietary patterns in health and disease. Nutrients 12(2):334 32012681 10.3390/nu12020334 PMC7071223

[6] Wang K, Niu Y, Lu Z, Duo B, Effah CY, Guan L (2023) The effect of breakfast on childhood obesity: a systematic review and meta-analysis. Front Nutr 10:1222536 37736138 10.3389/fnut.2023.1222536 PMC10510410

[7] Tognon G, Moreno LA, Mouratidou T, Veidebaum T, Molnár D, Russo P, Siani A et al (2014) IDEFICS consortium. Adherence to a mediterranean-like dietary pattern in children from eight European countries. The IDEFICS study. Int J Obes (Lond) 38(2):S108–S114 25219407 10.1038/ijo.2014.141

[8] Williams J, Buoncristiano M, Nardone P, Rito AI, Spinelli A, Hejgaard T et al (2020) A snapshot of European children’s eating habits: results from the fourth round of the WHO European Childhood Obesity Surveillance Initiative (COSI). Nutrients 12(8):2481 32824588 10.3390/nu12082481 PMC7468747

[9] Jaeger V, Koletzko B, Luque V, Gruszfeld D, Verduci E, Xhonneux A, Grote V (2023) Eating frequency in European children from 1 to 96 months of age: results of the childhood obesity project study. Nutrients 15(4):984 36839341 10.3390/nu15040984 PMC9958886

[10] Scaglioni S, De Cosmi V, Ciappolino V, Parazzini F, Brambilla P, Agostoni C (2018) Factors influencing children’s eating behaviours. Nutrients 10:706 29857549 10.3390/nu10060706 PMC6024598

[11] Bechthold A, Boeing H, Tetens I, Schwingshackl L, Nöthlings U (2018) Perspective: food-based dietary guidelines in Europe-scientific concepts, current status, and perspectives. Adv Nutr 9:544–560 30107475 10.1093/advances/nmy033 PMC6140433

[12] The European commission's science and knowledge service food-based dietary guidelines in Europe. https://knowledge4policy.ec.europa.eu/health-promotion-knowledge-gateway/topic/food-based-dietary-guidelines-europe_en. Accessed 26 June 2024

[13] Manios Y, Androutsos O, Lambrinou CP, Cardon G, Lindstrom J, Annemans L et al (2018) A school- and community-based intervention to promote healthy lifestyle and prevent type 2 diabetes in vulnerable families across Europe: design and implementation of the Feel4Diabetes-study. Public Health Nutr 21(17):3281–3290 30207513 10.1017/S1368980018002136 PMC10260800

[14] Anastasiou CA, Fappa E, Zachari K, Mavrogianni C, Van Stappen V, Kivelä J, Virtanen E, González-Gil EM, Flores-Barrantes P, Nánási A, Semánová C, Dimova R, Usheva N, Iotova V, Cardon G, Manios Y, Makrilakis K, Feel4Diabetes-study group (2020) Development and reliability of questionnaires for the assessment of diet and physical activity behaviors in a multi-country sample in Europe the Feel4Diabetes Study. BMC Endocr Disord 20(1):135 10.1186/s12902-019-0469-x PMC7066729 32164677

[15] Cole TJ, Lobstein T (2012) Extended international (IOTF) body mass index cut-offs for thinness, overweight and obesity. Pediatr Obes 7(4):284–294 22715120 10.1111/j.2047-6310.2012.00064.x

[16] Piqueras M, Campoy C, Miranda M, Emmett P (2014) Comparison of childhood size and dietary differences at age 4 years between three European countries. Eur J Clin Nutr 68:786–792 24781687 10.1038/ejcn.2014.43

[17] Giménez-Legarre N, Santaliestra-Pasías AM, Cardon G, Imre R, Iotova V, Kivelä J, Liatis S, Makrilakis K, Mavrogianni C, Milenkovic T, Nánási A, Tankova T, Timpel P, Willems R, Manios Y, Moreno LA, On Behalf of The Feel Diabetes-Study Group (2021) Cross-sectional associations between mothers and children’s breakfast routine-the feel4diabetes-study. Nutrients 13(3):720 33668380 10.3390/nu13030720 PMC7996176

[18] Monzani A, Ricotti R, Caputo M, Solito A, Archero F, Bellone S, Prodam F (2019) A systematic review of the association of skipping breakfast with weight and cardiometabolic risk factors in children and adolescents. What should we better investigate in the future? Nutrients 11:387 30781797 10.3390/nu11020387 PMC6412508

[19] Mahmood L, Flores-Barrantes P, Moreno LA, Manios Y, Gonzalez-Gil EM (2021) The Influence of parental dietary behaviors and practices on children's eating habits. Nutrients 13(4):1138 10.3390/nu13041138 PMC8067332 33808337

[20] Mahmood L, Moreno LA, Flores-Barrantes P, Mavrogianni C, Schwarz P, Makrilakis K, Liatis S, Cardon G, Willems R, Rurik I, Radó S, Tankova T, Iotova V, Usheva N, Manios Y, Gonzalez-Gil EM; Feel4Diabetes-Study Group (2022) Parental food consumption and diet quality and its association with children’s food consumption in families at high risk of type 2 diabetes: the Feel4Diabetes-study. Public Health Nutr 25(12):1–12 10.1017/S1368980022002245 PMC9991723 36217747

[21] Mahmood L, González-Gil EM, Schwarz P, Herrmann S, Karaglani E, Cardon G, De Vylder F, Willems R, Makrilakis K, Liatis S, Iotova V, Tsochev K, Tankova T, Rurik I, Radó S, Moreno LA, Manios Y, Feel4Diabetes-Study Group (2022) Frequency of family meals and food consumption in families at high risk of type 2 diabetes: the Feel4Diabetes-study. Eur J Pediatr 181(6):2523–2534 10.1007/s00431-022-04445-4 PMC9110493 35353229

[22] Ciardullo S, Salvatore MA, Mandolini D, Spinelli A, Bucciarelli M, Andreozzi S, Buoncristiano M, Nardone P on behalf of the 2008/9–2019 OKkio alla SALUTE Group (2023) Trend in Breakfast Consumption among Primary School Children in Italy. Nutrients 15(21):4632 10.3390/nu15214632 PMC10647676 37960286

[23] Brug J, van Stralen MM, Te Velde SJ, Chinapaw MJ, De Bourdeaudhuij I, Lien N et al (2012) Differences in weight status and energy-balance related behaviors among schoolchildren across Europe: the ENERGY-project. PLoS ONE 7(4):e34742 22558098 10.1371/journal.pone.0034742 PMC3338827

[24] Craveiro D, Marques S, Zvěřinová I, Máca V, Ščasný M, Chiabai A, Suarez C, Martinez-Juarez P, García de Jalón S, Quiroga S, Taylor T (2021) Explaining inequalities in fruit and vegetable intake in Europe: The role of capabilities, opportunities and motivations. Appetite 165:105283 33991644 10.1016/j.appet.2021.105283

[25] Amaral A, Carvalho A, Lima J (2022) Consumption of legumes in children from 3 to 6 years – evaluation of an intervention program. Eur J Public Health 3(ckac129):514

[26] Charneca S, Gomes AI, Branco D, Guerreiro T, Barros L, Sousa J (2023) Intake of added sugar, fruits, vegetables, and legumes of Portuguese preschool children: baseline data from smartFeeding4Kids randomized controlled trial participants. Front Nutr 10:1150627 37063316 10.3389/fnut.2023.1150627 PMC10090424

[27] Feraco A, Armani A, Amoah I, Guseva E, Camajani E, Gorini S, Strollo R, Padua E, Caprio M, Lombardo M (2024) Assessing gender differences in food preferences and physical activity: a population-based survey. Front Nutr 11:1348456 38445208 10.3389/fnut.2024.1348456 PMC10912473

[28] O’Neil CE, Nicklas TA, Zanovec M, Cho S (2010) Whole grain consumption is associated with diet quality and nutrient intake in adults: the National Health and Nutrition Examination Survey, 1999–2004. J Am Diet Assoc 110:1461–1468 20869484 10.1016/j.jada.2010.07.012

[29] Abdoli M, Scotto Rosato M, Cipriano A, Napolano R, Cotrufo P, Barberis N, Cella S (2023) Affect, body, and eating habits in children: a systematic review. Nutrients 15(15):3343 37571280 10.3390/nu15153343 PMC10420931

[30] Ortiz-Moncada R, Morales-Suárez-Varela M, Avecilla-Benítez Á, Norte Navarro A, Olmedo-Requena R, Amezcua-Prieto C, Cancela JM, Blázquez Abellán G, Mateos-Campos R, Valero Juan LF, Redondo Martín S, Alonso-Molero J, Molina de la Torre AJ, Llopis-Morales A, Peraita-Costa I, Fernández-Villa T, de Investigación G, UniHcos (2019) Factors Associated with Meat Consumption in Students of Spanish Universities: UniHcos Project. Int J Environ Res Public Health 16(20):3924 10.3390/ijerph16203924 PMC6843505 31619016

[31] Saarni K, Honkanen A, Setälä J (2008). Factors affecting fish consumption in finnish catering outlets. Available: https://ir.library.oregonstate.edu/downloads/sn009z60w. Accessed 27 May 2024

[32] Nicolau-Nos R, Pujol-Andreu J, Hernández I (2020) Milk, social acceptance of a new food in Europe: Catalonia, 19th-20th centuries. Dynamis 30:119–139 10.4321/s0211-95362010000100005 20695167

[33] Magriplis E, Michas G, Petridi E et al (2021) Dietary sugar intake and its association with obesity in children and adolescents. Children (Basel) 8(8):676 34438567 10.3390/children8080676 PMC8391470

[34] Jústiz AM, Landry MJ, Asigbee FM et al (2020) Associations between child and parent knowledge of added sugar recommendations and added sugar intake in multiethnic elementary-aged children. Curr Dev Nutr 4(9):1–8 32923924 10.1093/cdn/nzaa140 PMC7475003

[35] O’Connor TM, Yang SJ, Nicklas TA (2006) Beverage intake among preschool children and its effect on weight status. Pediatrics 118(4):e1010–e1018 17015497 10.1542/peds.2005-2348

[36] Vereecken CA, Inchley J, Subramanian SV, Hublet A, Maes L (2005) The relative influence of individual and contextual socio-economic status on consumption of fruit and soft drinks among adolescents in Europe. Eur J Public Health 15(3):224–232 15905182 10.1093/eurpub/cki005

[37] Gonzalez-Palacios S, Navarrete-Muñoz EM, García-de-la-Hera M, Torres-Collado L, Santa-Marina L, Amiano P, Lopez-Espinosa MJ, Tardon A, Riano-Galan I, Vrijheid M, Sunyer J, Vioque J (2019) Sugar-containing beverages consumption and obesity in children Aged 4–5 Years in Spain: the INMA study. Nutrients 11(8):1772 31374897 10.3390/nu11081772 PMC6722971

[38] Linardakis M, Sarri K, Pateraki MS, Sbokos M, Kafatos A (2008) Sugar-added beverages consumption among kindergarten children of Crete: effects on nutritional status and risk of obesity. BMC Public Health 2008(8):279 10.1186/1471-2458-8-279 PMC2525654 18684334

[39] Clifton PM, Chan L, Moss CL, Cobiac L (2011) Beverage intake and obesity in Australian children. Nutr Metab (Lond) 8:87 22152289 10.1186/1743-7075-8-87 PMC3286426

[40] Vereecken C, Pedersen TP, Ojala K, Krølner R, Dzielska A, Ahluwalia N, Giacchi M, Kelly C (2015) Fruit and vegetable consumption trends among adolescents from 2002 to 2010 in 33 countries. Eur J Public Health 25:16–19 25805780 10.1093/eurpub/ckv012

[41] Goldbohm RA, Rubingh CM, Lanting CI, Joosten KF (2016) Food consumption and nutrient intake by children Aged 10 to 48 months attending day care in the Netherlands. Nutrients 8(7):428 27428995 10.3390/nu8070428 PMC4963904

[42] Lage Barbosa C, Brettschneider AK, Haftenberger M, Lehmann F, Frank M, Heide K, Patelakis E, Perlitz H, Krause L, Houben R, Butschalowsky HG, Richter A, Kamtsiuris P, Mensink GBM (2017) Comprehensive assessment of food and nutrient intake of children and adolescents in Germany: EsKiMo II - the eating study as a KiGGS module. BMC Nutr 3:75 32153853 10.1186/s40795-017-0196-5 PMC7050737

[43] Turck D, Bohn T, Castenmiller J, de Henauw S, Hirsch-Ernst KI, Maciuk A, Mangelsdorf I, McArdle HJ, Naska A, Neuhauser-Berthold M, Nowicka G, Pentieva K, Siani A, Stern M, Tomé D, Vinceti M, Willatts P, Wolfram G, Engel KH (2022) Tolerable upper intake level for dietary sugars. EFSA J 20(2):e07074 35251356 10.2903/j.efsa.2022.7074 PMC8884083

[44] Zhang FF, Roberts SB, Must A, Wong WW, Gilhooly CH, Kelly MJ, Parsons SK, Saltzman E (2015) Assessing dietary intake in childhood cancer survivors: food frequency questionnaire versus 24-hour diet recalls. J Pediatr Gastroenterol Nutr 61(4):499–502 25883059 10.1097/MPG.0000000000000826 PMC4581885

[45] Anderson L, Benbow HM, Manzin G (2021) Europe on a plate: food, identity and cultural diversity in contemporary Europe. Australian New Zealand J European Stud 8(1):1–15

