# Supplementary material for: A snapshot of country-specific dietary habits and obesity in European children: the Feel4Diabetes study
Source: Eur J Pediatr. 2025 Feb 27;184(3):214. doi: 10.1007/s00431-025-06037-4 (PMC11868321; doi:10.1007/s00431-025-06037-4)
Supplement: Supplementary file 1 — (DOCX 19.7 KB) [file 431_2025_6037_MOESM1_ESM.docx]

**Table 1. The frequency of food consumption according to the Food-Based Dietary Guidelines (FBDGs) in Europe**

| **Foods and beverages** | **Servings** | **Dietary**  **Guidelines** |
| --- | --- | --- |
| Milk and milk products (no cheese) | < 2 servings/day | Not Met |
|  | 2-3 servings/day | Met |
|  | > 3 servings/day | Exceed |
| Grains (Bread and BF cereals only) ^*^ | < 3 servings/day | Not Met |
|  | 3-5 servings/day | Met |
|  | > 5 servings/day | Exceed |
| Fruits | < 2 servings/day | Not Met |
|  | 2-3 servings/day | Met |
|  | > 3 servings/day | Exceed |
| Vegetables | < 3 servings/day | Not Met |
|  | 3-4 servings/day | Met |
|  | > 4 servings/day | Exceed |
| Legumes | < 2 servings/week | Not Met |
|  | 2-3 servings/week | Met |
|  | > 3 servings/week | Exceed |
| Red meat | < 2 servings/week | Not Met |
|  | 2-3 servings/week | Met |
|  | > 3 servings/week | Exceed |
| White meat and poultry | < 2 servings/week | Not Met |
|  | 2-3 servings/week | Met |
|  | > 3 servings/week | Exceed |
| Fish and seafood | < 2 servings/week | Not Met |
|  | 2-3 servings/week | Met |
|  | > 3 servings/week | Exceed |
| Salty snacks | < 1 servings/week | Recommended |
|  | 1-2 servings/week | Accepted |
|  | > 2 servings/week | Exceed |
| Sweet snacks | < 1 servings/week | Recommended |
|  | 1-2 servings/week | Accepted |
|  | > 2 servings/week | Exceed |
| Sweetened beverages ^a^ | < 1 serving/week  1 serving/week  > 1 serving/week | Recommended Accepted  Exceed |

* BF: Breakfast (rice and pasta were not mentioned under Grains group in the questionnaire). ^a^ mentioned in the dietary guidelines as grams of sugar, not as portions or servings of products. N/A: Not Applicable.

1 serving of milk/milk products= 200 ml, 1 serving of grains= 30 g, 1 serving of fruits = 90 g, 1 serving of vegetables= 150-200 g, 1 serving of legumes= 150 g cooked, 1 serving of red meat/poultry= 100 g, 1 serving of fish= 100 g cooked, 1 serving of salty snacks= 100 gm, 1 serving sweets= 40 g, 1 serving of sweetened beverages= 1/4 litre (added sugar: max. 10% daily energy).

**Table 2. The frequency of food consumption according to the Food-Based Dietary Guidelines (FBDGs) in Europe**

| **Food groups** | | **Foods** |
| --- | --- | --- |
| Nutrient-dense foods | Green vegetables and beans |  |
|  | Other vegetables |  |
|  | Fruits |  |
|  | Skim milk and low-fat dairy products |  |
| Energy-dense, low-nutrient foods | Whole milk |  |
|  | Soda |  |
|  | Proceed foods in general | Crackers |
|  |  | Cookies |
|  |  | Pizza |
|  |  | Fried food items |
|  | Sweets | Deserts in general |
|  |  | Chocolate |
|  |  | Cake |
|  | Fatty/processed meats | Fried chicken |
|  |  | Sausage/salami |
|  | Fats |  |

Source: The European Food-Based Dietary Guidelines (FBDGs) [12].
